# Prevalence of *Blastocystis* and its association with *Firmicutes/Bacteroidetes* ratio in clinically healthy and metabolically ill subjects

**DOI:** 10.1186/s12866-021-02402-z

**Published:** 2021-12-11

**Authors:** Claudia Muñoz Yañez, Alejandra Méndez Hernández, Alondra Martínez Sandoval, María Aurora Maravilla Domínguez, Soraya Amalí Zavaleta Muñiz, Janeth Oliva Guangorena Gómez

**Affiliations:** 1 Laboratorio de Microbiología y Parasitología Molecular, Facultad de Ciencias de Salud, Universidad Juárez del Estado de Durango, Sixto Ugalde y Palmas I S/N, Col Revolución, C.P. 35050 Gómez Palacio, Durango Mexico; 2 Instituto de Ciencia y Medicina Genómica, Av. Juarez #1822, Primero de Cobián Centro, C.P. 27000 Torreón, Coahuila Mexico

**Keywords:** *Blastocystis*, *Firmicutes*, *Bacteroidetes*, *F/B* ratio, Subtypes

## Abstract

**Background:**

*Blastocystis* is a typical anaerobic colon protist in humans with controversial pathogenicity and has relation with alterations in the intestinal microbiota composition (dysbiosis), whose eventual indicator is the *Firmicutes/Bacteroidetes* ratio (*F/B* ratio); this indicator is also linked to complications such as diabetes, obesity, or inflammatory bowel disease. The present study investigated the prevalence of *Blastocystis* and its association with *Firmicutes/Bacteroidetes* ratio in healthy and metabolic diseased subjects.

**Methods:**

Fecal and blood samples were collected consecutively from 200 healthy subjects and 84 subjects with metabolic disease; *Blastocystis* and its most frequent subtypes were identified by end-point PCR and the two most representative phyla of the intestinal microbiota *Firmicutes* and *Bacteroidetes* by real-time PCR.

**Results:**

The prevalence of *Blastocystis* in healthy subjects was 47.0, and 65.48% in subjects with metabolic disease; the most prevalent subtype in the total population was ST3 (28.38%), followed by ST1 (14.86%), ST4, ST5, and ST7 (each one of them with 14.19% respectively), and finally ST2 (8.78%). The low *F/B* ratio was associated with the prevalence of *Blastocystis* in the two cohorts FACSA (OR = 3.78 *p* < 0.05) and UNEME (OR = 4.29 *p* < 0.05). Regarding the subtype level, an association between the FACSA cohort ST1 and ST7 with low *Firmicutes/Bacteroidetes* ratio was found (OR = 3.99 and 5.44 *p* < 0.05, respectively).

**Conclusions:**

The evident predatory role of *Blastocystis* over *Firmicutes* phylum was observed in both cohorts since the abundance of bacterial group’s *Bacteroidetes* increases in the groups colonized by this eukaryote and, therefore, may have a beneficial effect.

**Supplementary Information:**

The online version contains supplementary material available at 10.1186/s12866-021-02402-z.

## Background

The population of microorganisms living in the human body, especially in the gut and its communication, is termed microbiota [1]. About 10^14^ bacterial cells live in the colon besides viruses and eukaryotic microorganisms [1, 2], which participate in human metabolism interrelatedly [3]. The gut microbiota plays a crucial role in maintaining the host’s physiological functions; disrupting the fragile host-microbiota interaction equilibrium could affect the onset of several metabolic diseases [4]. Recently, this disruption has been implicated in some chronic diseases ranging from inflammatory bowel disease (IBD), type 2 diabetes mellitus (T2DM), and cardiovascular disease (CVD) to colorectal cancer [5].

The two predominant phyla *Firmicutes* and *Bacteroidetes,* are determined mainly by the characteristics of the diet and by some genetic and environmental factors that influence the predominance of some organisms over others [6]. In case of an imbalance in the microbiota, dysbiosis will occur [7]. The modification in *Firmicutes/Bacteroidetes* ratio is an eventual [8] indicator of changes in the microbiota’s composition, which leads to the development of long-term complications such as obesity, diabetes, or inflammatory bowel disease [9, 10]. Noteworthy, the measure of the *Firmicutes/Bacteroidetes* ratio is a rough method to characterize the microbiota and should note that it is an analysis at the phylum level, which does not have a high resolution from the taxonomic point of view [11]. More standardized and accurate procedures are needed to compare studies from different laboratories and a more taxonomically detailed description than phylum-level changes [8]. Several mechanisms have been proposed to describe the intestine’s changes and energy metabolism from diets rich in carbohydrates and fats [12] that condition the gut microbiota’s dysbiosis, showing a *Firmicutes* phylum predominance in individuals with obesity and type 2 diabetes mellitus [13, 14].

Conversely, some parasites can colonize the human intestine causing changes in the typical microbiota composition. *Blastocystis* is the most frequent enteric protozoa found in the human intestine and various animals; however, its clinical significance remains controversial [15]. Infections by *Blastocystis* range from asymptomatic carriage to non-specific gastrointestinal symptoms; besides, it has been linked to irritable bowel syndrome and urticaria in some populations [16]. This protozoan can inhabit the human intestine for long periods without causing symptoms, so it can probably be part of the normal intestinal microbiota, perhaps its controversial role is mainly due to the predominant subtype or the association with viruses and bacteria [17, 18]. Currently, 22 subtypes (STs) have been identified, 17 have been recognized, and the rest are still under investigation; in humans, only ten have been identified (ST1-ST9 and ST12) [19].

The presence of *Blastocystis* increases the diversity of gut bacteria [20, 21], and healthier individuals often harbour greater gut microbiological diversity [22]. A negative correlation between *Blastocystis* and body mass index (BMI) has been suggested [23]; however, it is unknown whether the bacterial microbiota of these individuals induces colonization by *Blastocystis* or the presence of this protozoan promotes specific bacterial microbiota in individuals with low BMI [21, 24]. *Blastocystis* may cause a low *Firmicutes/Bacteroidetes* ratio and gastrointestinal symptoms, and even has been extensively studied in populations with irritable bowel syndrome and disease, the associations found are not strong enough to be attributed to the parasite [25, 26]. Currently, it is unknown whether *Blastocystis* participates in changes in the microbiota or changes in microbiota and metabolic dysfunctions that cause high colonization by this protozoan [27]. However, a higher relative abundance of *Bacteroidetes* and a decrease in *Firmicutes* does not necessarily respond to beneficial effects. Although, it has been demonstrated an increase in *Firmicutes* (*Faecalibacterium* and *Clostridia* family) in individuals with *Blastocystis* [20, 26] and a decrease in *Bacteroidetes* related to healthy individuals [28], or in patients with *Clostridium difficile* colonized by *Blastocystis* [29]; the association with alterations of the microbiota in metabolically ill subjects is unknown. This study aimed to evaluate the prevalence of *Blastocystis*, subtypes and its association with low *Firmicutes/Bacteroidetes* ratio in clinically healthy and metabolically ill subjects.

## Results

Two hundred two young university students (FACSA cohort) were included (two were eliminated due to insufficient samples). Of 95 metabolically ill adults (UNEME cohort), 11 were eliminated due to bad samples and incomplete questionnaires. The subjects of the FACSA cohort were younger than the UNEME cohort; the median age in the groups was 20 (19–21) vs. 55 (46–63) years, respectively (Mann-Whitney-Wilcoxon test *p* = 0.0001) respectively. The subjects of the UNEME cohort have a higher body mass index than the FACSA cohort. The percentage of subjects with normal BMI is higher in the FACSA cohort, and the urban area is the most represented in the total sample. Still, in sick adults, the rural area is more representative (Table 1).

**Table 1:** Characterization of FACSA and UNEME cohort

	Total	FACSA	UNEME
*Age, years*	21 (19–45)	20 (19–21)	55 (46–63)^a^
*Sex, n(%)*			
*Female*	201 (70.77)	132 (66.0)	69 (82.14)^a^
*Male*	83 (29.23)	68 (34.0)	15 (17.86)
*BMI, kg/mt2*	26.60 (22.39–31.4)	24.29 (21.08–28.76)	31.55 (28–35.50)^a^
*Normal*	118(41.55)	111(55.50)	7 (8.33)^a^
*Overw/obesity*	166(58.45)	89(44.50)	77(91.67)
***Place of residence***			
*Urban*	197 (69.37)	170 (85.0)	27 (32.14)
*Rural*	87 (30.63)	30 (15.0)	57 (67.86)^a^
***Hematic Biometry***			
Leukocytess/mm3	6.91 ± 1.67	6.61 ± 1.60	7.57 ± 1.63^a^
Total Lymphocytes/mm3	2.2 (1.8–2.6)	2.1 (1.7–2.5)	2.3 (1.9–2.7)^a^
Total MXD /mm3	0.5 (0.4–0.6)	0.5 (0.4–0.6)	0.5 (0.3–0.7)^a^
Total Neutrophils /mm3	4.10 ± 1.3	3.92 ± 1.26	4.65 ± 1.31^a^
Platelets/mm3	257.44 ± 57.95	250.84 ± 51.64	272.44 ± 68.1^a^

*BMI:* Body mass index, *kg:* Kilograms, *mt*^*2*^: Square meter, *Overw/obesity* : Overweight and obesity, *n:* Number, *(%)* :Percentage, *MXD:* Monocytes, eosinophils and basophils count, *mm3:* Cubic millimeter. x^2^, Mann-Whitey-Wilcoxon, *t* de student, ^a^: *p*<0.05

### Prevalence of *Blastocystis* and subtypes

*Blastocystis* was more prevalent in the UNEME cohort 55 (65.48%) vs. 94 (47.0%) (x^2^ test, *p =* 0.004*)*. For subtypes, ST5 was the most prevalent in the UNEME cohort 16 (29.63%) (x^2^ test, *p* = 0.001) while ST4 was more prevalent in the FACSA cohort, although the data are not statistically significant (Table 2).

**Table 2:** Prevalence of *Blastocystis* and subtypes, gut intestinal in FACSA and UNEME cohort

	Total	FACSA	UNEME
***Blastocystis*, n (%).**	153 (52.46)	94 (47.0)	55 (65.48)*
Subtype 1 (ST1)	22 (14.86)	14 (14.89)	8 (14.81)
Subtype 2 (ST2)	14 (8.78)	8 (8.51)	5 (9.26)
Subtype 3 (ST3)	42 (28.38)	28 (29.79)	14 (25.93)
Subtype 4(ST4)	21 (14.19)	16 (17.02)	5 (9.26)
Subtype 5 (ST5)	21 (14.19)	5 (5.32)	16 (29.63)*
Subtype 7 (ST7)	21 (14.19)	12 (12.77)	9 (16.67)
***Gut microbiota***			
RAUF, Median IR	1.07 (0.23–2.35)	0.80 (0.05–2.08)	1.92 (0.71–3.53)*
RAUB, Median IR	0.96 (0.47–2.11)	0.82 (0.44–1.65)	1.16 (0.63–3.29)*
*F/B* ratio, Median IR	0.95 (0.22–3.50)	0.83 (0.07–3.50)	1.40 (0.32–4.03)*

*n* Number, *(%)* Percentage, *IR* Interquartile range, *RAUF* Relative abundance units *Firmicutes*, *RAUB* Relative abundance units *Bacteroidetes*, *F/B ratio Firmicutes/Bacteroidetes* ratio. x^2^, Mann-Whitney-Wilcoxon

* *p* < 0.05

Relative abundance units *Firmicutes* (RAUF), Relative abundance units *Bacteroidetes* (RAUB), and *Firmicutes/Bacteroidetes* ratio (*F/B* ratio).

The RAUF was higher in UNEME cohort 1.92 (0.71–3.53) vs. FACSA cohort 0.80 (0.05–2.08), similar results were obtained with the RAUB 1.6 (0.63–3.29) vs. 0.82 (0.44–1.65), and *F/B* ratio 1.40 (0.32–4.03) vs. 0.83 (0.07–3.50) (Mann-Whitney-Wilcoxon test, *p* = 0.0001, *p* = 0.005, and *p* = 0.031, respectively) (Table 2).

### Prevalence of *Blastocystis* and their association con *Firmicutes/Bacteroidetes* ratio

**Fig. 1:**
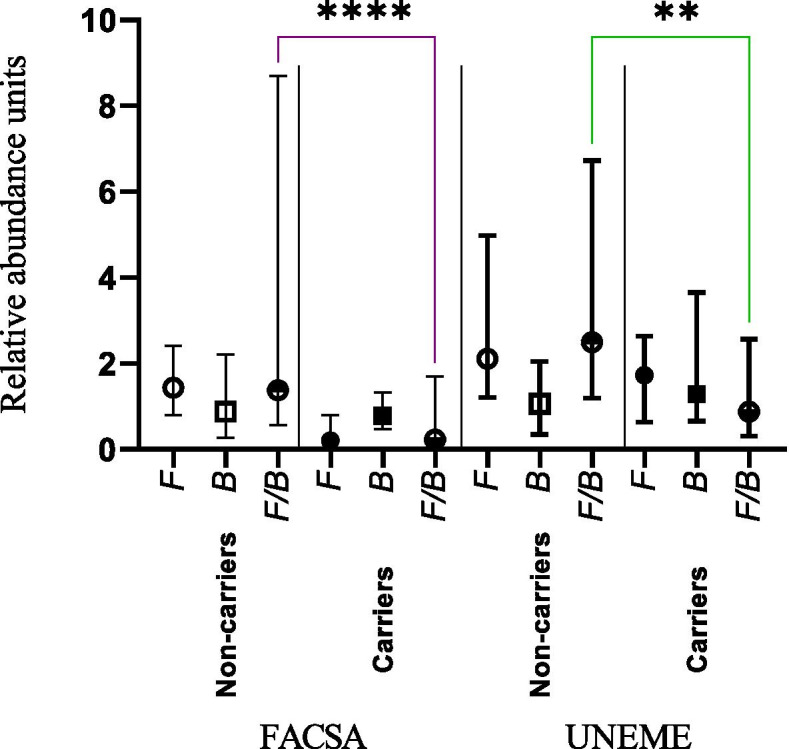
Relative Abundance Units of *Firmicutes* (F), *Bacteroidetes* (B) and *Firmicutes / Bacteroidetes* Ratio (*F / B*) in non-carriers and carriers by *Blastocytsis* of the FACSA and UNEME cohort. The *F/B* Ratio was decreased in subjects colonized by *Blastocystis* in both cohorts. (Mann-Whitney-Wilcoxon test, *p* = 0.0001, and *p* = 0.015, respectively)

The prevalence of *Blastocystis* was associated with a lower *F/B* ratio both in the FACSA cohort and in the UNEME cohort, 0.23 (0.02–1.6) carriers vs. 1.3 (0.5–8.2) no carriers, and 0.88 (0.31–2.5) carriers vs. 2.4 (1.2–6.1) no carriers, (Mann-Whitney-Wilcoxon test, *p* = 0.0001, and *p* = 0.015, respectively) (Fig. 1). This association was also observed with subtypes 1,2,4 and 7 in the FACSA cohort, (Mann-Whitney-Wilcoxon test, *p* = 0.003, *p* = 0.027, *p* = 0.006 and *p* = 0.0007, respectively) (Fig. S1: B, C, E and G). The prevalence of *Blastocystis* was not associated with age, sex, or obesity in either two cohorts, Table 3. Regarding the ST2 and ST3 subtypes, were associated with age in the UNEME cohort, the oldest patients are those colonized by these subtypes (Mann Whitney-Wilcoxon test *p* = 0.043 and *p* = 0.039, respectively) (Table S7).

**Table 3:** Prevalence of *Blastocystis* and its association with age, sex, hematologic parameters and gut microbiota in FACSA and UNEME cohort

	*FACSA Blastocystis*		*UNEME Blastocystis*	
	no carriers	carriers	no carriers	carriers
Age, median IR	20 (18–21)	20 (19–21)	52 (47–61)	57 (46–64)
**Sex, n(%)**				
Male	35 (51.4)	33 (48.5)	6 (40.0)	9 (60.0)
Female	71 (53.7)	61 (46.2)	23(33.3)	46 (66.6)
Normal	60 (54.0)	51 (45.9)	1(14.2)	6 (85.7)
Overweight/obesity	46 (51.6)	43 (48.3)	29 (34.5)	49 (63.6)
***Place of residence***				
*Urban*	89 (83.93)	81 (86.17)	11 (37.93)	16 (29.09)
*Rural*	17 (16.04)	13 (13.83)	18 (62.07)	39 (70.91)
***Hematic Biometry***				
Leukocytes/mm3	6.59 ± 1.54	6.64 ± 1.59	7.53 ± 1.40	7.59 ± 1.69
Total Lymphocytes/mm3	2 (1.7–2.5)	2.2 (1.8–2.7)	2.3 (1.9–2.7)	2.4 (2–2–7)
Total MXD /mm3	0.5 (0.4–0.6)	0.5(0.4–0.6)	**0.35 (0.2–0.5)**	0.5 (0.3–0.7)*
Total Neutrophils /mm3	3.91 ± 1.27	3.92 ± 1.28	4.63 ± 1.16	4.73 ± 1.45
Platelets/mm3	**257.51 ± 51.05**	242.40 ± 51.55*	280.13 ± 67.68	270.07 ± 70.44
**Microbiota**				
RAUF	1.4 (0.80–2.8)	0.26 (0.05–0.7)*	2.1 (1.2–4.9)	1.7 (0.63–2.2)
RAUB	0.88(0.28–2.1)	0.78(0.47–1.3)	1.06(0.35–1.9)	1.2(0.65–3.6)
Ratio F/B	1.3(0.5–8.2)	0.23(0.02–1.6)*	2.4(1.2–6.1)	0.88(0.31–2.5)*

*IR* Interquartile Range, *n* Number, *%* Percentage, *mm3* Cubic millimeter, *RAUF* Relative abundance units of *Firmicutes*, *RAUB* Relative abundance units of *Bacteroidetes*, *F/B ratio Firmicutes/Bacterioidetes*, Mann-Whitney-Wilcoxon test, *x*^*2*^ Chi-square test

*p < 0.05

We considered for the logistic regression analysis a high *F/B* ratio > 0.83 and low *F/B* ratio < 0.83 in the FACSA cohort; and a high *F/B* ratio > 1.40 and low *F/B* ratio < 1.40 in the UNEME cohort according to the results of Table 2. The association was corroborated with respect to *Blastocystis* OR = 3.78 (95% CI 2.10–6.81 *p* = 0.0002) and OR = 4.24 (95% CI 1.59–11.31 *p* = 0.002) (Tables 4 and 5, respectively), ST1 and ST7 were associated with low *Firmicutes/Bacteroidetes* ratio in the FACSA cohort but not in the UNEME cohort, ST1 has OR = 3.99 (CI 95% 1.07–14.79 *p* = 0.039) and ST7 OR = 5.44 (95% CI 1.16–25.52 *p* = 0.022) (Table 4).

**Table 4:** *Blastocystis*, subtypes and their association with Low *Firmicutes/Bacteroidetes* ratio in FACSA cohort

	OR	CI 95%	*P-value*
*Blastocystis*	3.78	(2.10–6.81)	**0.00**
ST1	3.99	(1.07–14.79)	**0.03**
ST2	3.12	(0.61–15.88)	0.16
ST3	1.97	(0.86–4.52)	0.10
ST4	2.34	(0.78–7.02)	0.12
ST5	4.12	(0.45–37.57)	0.20
ST7	5.44	(1.16–25.52)	**0.03**

*OR* Odds Ratio, *CI 95%* Confidence interval to 95%. *p* < 0.05

**Table 5:** Logistic regression between *Blastocystis* and subtypes and its association with low *Firmicutes/Bacteroidetes* ratio in a UNEME cohort

	OR	CI 95%	*P-value*
***Blastocystis***	**4.24**	**(1.59–11.31)**	**0.00**
ST1	0.58	(0.13–2.62)	0.48
ST2	1.57	(0.24–9.97)	0.62
ST3	3.06	(0.87–10.72)	0.08
ST4	0.23	(0.02–2.22)	0.20
ST5	2.71	(0.84–8.66)	0.09
ST7	2.22	(0.51–9.58)	0.28

*OR* Odds Ratio, *CI 95%* Confidence interval to 95%. *p* < 0.05

## Discussion

Our most important findings are the higher *Blastocystis* prevalence in the UNEME cohort than in the FACSA cohort (Table 2). The faecal-oral transmission of *Blastocystis* is due to poor hygiene practices, exposure to animals infected with the parasite, and intake of contaminated water or food [30]. This transmission route is probably most common since the most UNEME cohort subjects live in rural areas (Table 1).

The *F/B* ratio comparison between cohorts showed an *F/B* ratio high in UNEME cohort subjects (Table 2) according to a high *F/B* ratio (Median of 1.40; IR: 0.32–4.03) compared to the subjects of the FACSA cohort (Median of 0.830; IR: 0.07–3.50, *p* < 0.05). A high *F/B* ratio agrees with previous reports, in which it is found in metabolically ill subjects [4]; this relationship implies a predisposition to disease states [31]. Likewise, a low *Firmicutes/Bacteroidetes* ratio is related to weight loss [32], which corresponds with FACSA cohort individuals with lower BMI in this study (Table 1).

Obese and diabetic individuals, compared to healthy individuals, have a higher relative abundance of *Firmicutes* and a reduced abundance of *Bacteroidetes* [13], as well as a low microbial gene count and a dominance in the genera *Bacteroides* and *Ruminococcus*, all this is associated with a more remarkable ability to obtain energy from the diet, systemic inflammation, adiposity, insulin resistance, and dyslipidemia [22, 33]. *Bacteroidetes* are known to produce mainly acetate and propionate, while *Firmicutes* produce more butyrate, attributed to anti-inflammatory activities, regulation of energy metabolism, and increases leptin [34, 35].

The increase in *Firmicutes* in metabolically ill patients could cause an increase in butyrate production leading to antiobesogenic effects, which is contradictory. It has been speculated that in obese subjects, the butyrate-producing bacteria decrease and are replaced by other bacteria belonging to the same phylum resulting in lower butyrate production in the colonic lumen [8]. For example, increased abundances of *Staphylococcus* spp. and *Lactobacillus reuteri* (both from the phylum *Firmicutes*) have been reported in obese people and positively correlated with energy intake and plasma C-reactive protein (CRP), respectively [36, 37]. On the contrary, the decreased abundance of the butyrate-producing *Faecalibacterium prausnitzii* (phylum *Firmicutes*) correlated negatively with the intensity of low-grade inflammation in obese subjects and type 2 diabetes patients [38, 39].

*Blastocystis* is a widely distributed organism with great adaptability that could colonize healthy and diseased subjects. We analyzed the *Firmicutes/Bacteroidetes* relationship and the presence of *Blastocystis* in these two cohorts, and the results showed an association with a low *F/B* ratio (Table 3) (Fig. 1).

The relationship of *Blastocystis* with the gut microbiota is a subject of debate, as it has been linked to a low *F/B* ratio and irritable bowel disease [26]. However, Audebert et al. [20] found a greater abundance of Clostridiales at the class level and a greater abundance of *Rumminococcaceae* and *Prevotellaaceae* at the family level in subjects with *Blastocystis*, while *Enterobacteriaceae* increased in patients without *Blastocystis*. It has been suggested that *Blastocystis* is not associated with dysbiosis observed in intestinal, metabolic diseases or infections commonly associated with inflammation of the lower gastrointestinal tract; instead, colonization by this protozoan could be associated with a healthy intestinal microbiota [20].

Since previous studies that have found a high *Firmicutes / Bacteroidetes* ratio in healthy patients when *Blastocystis* is present, a study in faecal samples from 35 Swedish university students found that the presence of *Blastocystis* was accompanied by higher abundances of the bacterial generates Sporolactobacillus. Also, *Blastocystis* carriage was positively associated with high bacterial genus richness and negatively correlated to the Bacteroides-driven enterotype; although the results were not significant, the associations between *Blastocystis* and the bacterial microbiota found in this study could imply a link between *Blastocystis* and a healthy microbiota as well as with diets high in vegetables [28].

Another study carried out in stool samples from 57 school-aged children in Colombia found a higher microbial richness in *Blastocystis*-colonized children, which could be benefit intestinal health. The phylum *Firmicutes* was the predominant taxonomic unit in both groups analyzed; nevertheless, the composition of the intestinal bacterial community was not significantly different between *Blastocystis*- free and *Blastocystis*-colonized children [40]. It is worth mentioning that diet [41, 42], antibiotics [43], age, geographic area [44], inflammation [45] and, to a lesser extent, genetics of the host [46] affect the ecology of the intestinal bacterial community. On the other hand, the heterogeneity of the results in humans, regarding the *Firmicutes/ Bacteroidetes* ratio, could be due to the insufficient number of subjects included in most of the studies, making a poor statistical power to detect small variations [47].

Regarding experimental studies in a study, reported the decrease in the *Firmicutes/Bacteroidetes* ratio (*p* = 0.06) [48] in a model of IBS (Irritable Bowel Syndrome) in rats infected with *Blastocystis* ST4 from healthy humans. This partially agrees with our findings since in the FACSA cohort ST2 had an OR = 3.12 (0.61–15.88, 95% CI), ST3 OR = 1.97 (0.86–4.52, 95% CI), while ST4 OR = 2.34 (0.78–7.02, 95% CI), *p* > 0.05 (Table 4) was not associated with low *F/B* ratio. However, there was an association with low *F/B* at the gender level OR = 3.78 (2.10–6.81, 95% CI), for the subtypes ST1 OR = 3.99 (1.07–14.79, 95% CI) and ST7 OR = 5.44 (1.16–25.52, 95% CI) *p* < 0.05, (Table 4). Regarding ST7, Yason et al. [49] reported that the presence of ST7 decreases the *Bifidobacterium* and *Lactobacillus* populations while increasing the *Escherichia* populations.

About ST1, the presence of an alternative oxidase provides a partially dependent metabolism of molecular oxygen to resist the stress that the high oxygen concentration entails, which may occur in the intestine of the FACSA subjects due to the low *F/B* ratio [50]. Also, in the UNEME cohort, a low *F/B* ratio was found concerning gender OR = 4.24 *p* < 0.05 (Table 5) but not for subtypes.

The low *F/B* ratio found in this study might imply that the subjects of the two cohorts infected by *Blastocystis* present gastrointestinal symptoms; however, the association of the *Blastocystis* prevalence with gastrointestinal symptoms showed an inverse association between abdominal pain and ST1. At the same time, ST4 was inversely associated with abdominal distension in the FACSA cohort in previous data published [51] (Tables S2 and S3); however, no association of *Blastocystis* with gastrointestinal symptoms was found in the UNEME cohort (Tables S4 and S5). Contrary to the results obtained, a study found that *Blastocystis* negatively correlates with *Bacteroidetes* [20], while the phylum *Firmicutes* presents a positive correlation in *Blastocystis* positive samples [24]. These results could be biased in type 2 diabetes subjects due to the low carbohydrate consumption [52]. Since *Firmicutes* have more coding genes for enzymes involved in metabolism, a decrease in carbohydrate consumption could lead to *Firmicutes* decrease and *Bacteroidetes* increase [13, 53]; however, we did not analyse diet in this study.

Possibly, a high *F/B* ratio does not always lead to inflammation or disease, but certain conditions that lead to an inflammatory state would have to be present to affect the individual. For example, in obese individuals, the proportion of *Firmicutes* and *Proteobacteria* increases, compared to *Bacteroidetes*; in this case, an inflammatory environment is present [54]. In the UNEME cohort, we observed *Firmicutes/Bacteroidetes* ratio increase in the UAR of *Bacteroidetes*, but this does not necessarily imply inflammation. Additionally, there is evidence that *Blastocystis* modulates the immune system through IL-22 release that stimulates mucus production, alleviates colitis symptoms [55] and induces an immune response with a predominance of the Th2 cell response, favouring an anti-inflammatory environment [56].

Interestingly, ST5 was more prevalent in the UNEME cohort. The detection of this subtype is not common in humans and, although low frequencies of ST5 have been reported in Bolivia, Pakistan, Thailand, the United Kingdom, Colombia, and China [18, 57–61], there are no reports of this subtype in Mexico [62]. Additionally, this subtype is mainly detected in non-human primates, pigs, ostriches, dogs, rats, and some ungulate mammals; and therefore, a zoonotic profile has been attributed to it [63, 64]. The above agrees with our findings since most of the subjects of the UNEME cohort are from rural areas (Table 1). ST5 may survive in the possible inflammation scenario, judging by the high *Firmicutes/Bacteroidetes* ratio of the UNEME cohort (Tables 1 and 2). ST5 could enrich different groups of bacteria while reducing competition between other bacterial communities of the microbiota.

Regarding ST4, although the prevalence was not high, it was more frequent in the FACSA cohort (clinically healthy subjects) subjects with lower BMI (Tables 1 and 2) and subjects with lower BMI in the total sample (Table S6). These findings agree with Beghini et al. [24] that found a strong negative correlation between BMI and *Blastocystis* prevalence. Also, consistent with findings from the Danish’s subjects study [23], the difference in *Blastocystis* prevalence between average weight and obese subjects (*p* = 5E-03), average weight and overweight (*p* = 0.01), and between non-overweight and overweight (*p* = 0.02) was significant. Between specific subtypes, only ST4 reached statistical significance (*p* = 0.03 between average weight and obese). Besides, Tito et al. [21] found a positive and significant correlation (*R* = 0.26 *p* = 0.00028) between ST4 and *Akkermansia* and *Methanobrevibacter*; the first is an abundant bacterium in healthy people that degrades intestinal mucin, which is associated with weight loss; the second is a *methanogenic archaeon* that plays an essential role in carbohydrate digestion and may protect against weight gain [22].

ST3 was the most prevalent in the FACSA cohort, 29.79%, and the second most prevalent, 25.93%, in the UNEME cohort (Table 2). This subtype was not associated with an intestinal low *F/B* ratio in either of the two cohorts (Tables 4 and 5), agreeing with previous reports [58, 65, 66]. A higher bacterial diversity has been reported in ST3-*Blastocystis*-carriers (high abundance of *Prevotella*, *Methanobrevibacter*, and *Ruminococcus*), while a high percentage of *Bacteroides* found in *Blastocystis*-free subjects [66]. Asnicar F. et al. [67] reported interesting findings of the presence of *Prevotella copri* and *Blastocystis* spp. as markers of improved postprandial glucose response; both were strongly linked with favourable glucose homeostasis and a decrease of the estimated visceral adipose tissue mass.

One of the limitations of our study was the analysis at the phylum level since it does not have a high resolution from the taxonomic point of view. However, the advantages of using a small template are the high sensitivity, high-performance processing and affordable cost [11]. And it is used in research that aims to characterize microbial communities [68]; such is the case in our study. In future studies, we will perform ultra-high-throughput sequencing methods. Also in future studies, we will analyse the dietary habits and the microbiota composition, including *Blastocystis*, in both cohorts. Another limitation of our work was qualitative PCR, which only identifies the presence or absence of ST. The implementation of a more sensitive molecular technique, such as Next-generation amplicon sequencing (Maloney (2019), could give us additional information, such as the most predominant ST or the existence of more than two subtypes in a single sample [69]. Also, this technique could help with the identification of genotypes that were not detected with the primers used. A more accurate assessment of *Blastocystis* diversity is key to understanding the transmission mechanism and pathogenicity in our population. Another limitation was that the majority of the subjects in the UNEME cohort were obese, and the analysis of the comparison with thin individuals between the two cohorts could not be carried out. Therefore, the objective is to increase the sample size in this cohort concerning this group.

## Conclusions

The present study provides an overview of the two most representative phyla behaviour, the intestinal microbiota *Firmicutes* and *Bacteroidetes* and the *Firmicutes/Bacteroidetes* ratio when *Blastocystis* is present. The modulation caused by the parasite mainly in the *Firmicutes* phylum is evident, which decreases in two cohorts. Some studies have reported the variability between subtypes but have not focused on the host’s variability. Based on this, it is interesting to analyze the type of diet since this may favour the colonization and the predatory function of *Blastocystis* in the intestine; additionally, follow-up studies will be carried out in the two cohorts.

## Methods

### Subjects and sample collection

This study was a cross-sectional design with a nonprobabilistic sampling conducted between March of 2018 to April 2019. Two hundred clinically healthy university young adults (FACSA cohort) and 84 adults with metabolic disease were included (UNEME cohort). Inclusion criteria for UNEME cohort were diabetes, hypertension, dyslipidemia, and overweight/obesity. Exclusion criteria were kidney failure, heart disease, lung disease, amputation, and pregnancy.

Inclusion criteria for the FACSA cohort were Bachelor students of the medicine and nutrition program. In this group, patients with chronic degenerative diseases were excluded.

Exclusion criteria for both groups were patients who received any medication with antibiotic treatment in the last 3 months before the study.

Data of patients with insufficient sample or incomplete information were eliminated.

### Questionnaire survey

A digital gastrointestinal symptoms questionnaire (abdominal pain and constipation), consisting of multiple-choice questions, based on Rome III diagnostic criteria [70, 71], was used to collect information about each participant including sex, age, city of origin, and clinical data. All data collected from each subject remained confidential and were fully anonymized through the encryption of the identity of individuals.

A 5 ml blood sample was taken of all individuals to perform hematic biometry and requested a stool sample to identify the intestinal microbiota and the presence of *Blastocystis.*

### *Blastocystis* identification

#### Parasitological examination

Samples were collected in containers with 10% formaldehyde for coproparasitological exams in triplicate. Each microscopic identification of *Blastocystis sp* was carried out on a different day of the deposition. The sample preparation was developed as described previously [51]. Briefly, the modified Ritchie technique was performed for the preparation of the samples. Ten microliters of each stool sample were mixed with 20 μl of Lugol’s iodine solution and covered with a 21 × 26 mm coverslip. Three hundred optical fields were examined for *Blastocystis* with a magnification of 250× (20× objective and 12.5 eyepieces) and, in case of suspected organisms, 500 × (40 × 12.5×) magnification. The observation of each slide lasted an average of 5 min. The diagnostic criterion for positivity was at least 2 precise vacuolar forms of the parasite in either of the three samples [72].

#### DNA extraction

To confirm the microscopy diagnosis, molecular biology techniques were used to detect *Blastocystis* subtypes. A fresh sample was dispensed into a DNase and RNAse-free sterile bottle and kept under refrigeration until transport to the laboratory, stored at − 20 °C until use. The sample extraction was developed as described previously [51]. Briefly, from 200 mg of faeces, the nucleic acid extraction was carried out using the E.Z.N.A.® Stool DNA Kit (USA). DNA concentration and purity were determined using NanoDrop 1000 Thermo Scientific (Saveen Werner ApS®, Denmark).

#### Genus determination

The extracted DNA samples were used to determine the presence of *Blastocystis*. Three microlitres of each DNA sample were mixed with Radiant™ Red 2x Taqman Mastermix (Alkali Scientific Inc.) to a final volume of 13 μl for the PCR. The primers used were: F1- 5′-GGA GGT AGT GAC AATAAA TC-3′ and R1- 5′-CGT TCA TGA TGA ACA ATT AC-3′ [73] (T4 Oligo®, Irapuato, México). All samples underwent PCR test.

### Subtyping of *Blastocystis* using sequence-tagged sites (STS) primers

For the genotyping of *Blastocystis (ST1-ST5, ST7)*, a set of sequence-tagged site primers derived from products of randomly amplified polymorphic DNA (RAPD) sequences were used [73, 74]. Four μl of each DNA sample positive for *Blastocystis* in a Polymerase chain reaction (PCR) was mixed with Radiant™ Red 2x Taqman Mastermix (Alkali Scientific Inc.) with primers (Table S1) in a final volume of 13 μl.

The PCR conditions were an initial denaturation step at 94 °C for 4 min; followed by 35 denaturation cycles at 94 °C for 30 s; annealing at 55 °C for 45 s; extension at 72 °C for 45 s; and a final extension at 72 °C for 10 min (PTC-100 thermocycler, MJ Research Inc) [30]. The ß-globin gene was amplified as an internal extraction control. The samples that were negative for gender but beta-globin positive underwent subtyping. The PCR products were resolved in a 1.5% agarose gel (Ultrapure Agarose, Invitrogen™) stained with RedGel™ Nucleic Acid Gel Stain (Biotium), and a molecular weight marker was used to establish the size of the amplicon (100 bp DNA Ladder. Biobasic Inc.). Additionally, the samples were randomized to the analysis by PCR. Sanger sequencing was used to corroborate both the presence of *Blastocystis* and genotypes, contrasted them with sequences reported in https://blast.ncbi.nlm.nih.gov/Blast.cgi using a blast. The nucleotide sequences generated in present study have been deposited in GenBank (https://www.ncbi.nlm.nih.gov/) under accession numbers: MZ351752-57.

### Identification of the gut microbiota

The analysis of the microbiota profile was performed by real-time PCR (qPCR) using the 16S rRNA taxon-specific to detect the presence of *Bacteroidetes/Firmicutes* and universal primers to amplify all members of the taxon. The sequences of the primers were: *Bacteroidetes*: CRAACAGGATTAGATACCCT (Forward) and GGTAAGGTTCCTCGGCTAT (Reverse); *Firmicutes*: TGAAACTYAAGGAATTGACG (Forward) and ACCATGCACCACCTGTC (Reverse); universal: AAACTCAAAKGAATTGACGG (Forward) and CTCACRRCACGAGCTGAC (Reverse) [11] (T4 Oligo®, Irapuato, México). The specificity of the amplification products and the absence of primer dimers were determined by performing melting curve analyses in all cases. The standard curve for each primer was generated by 5-fold serial dilutions of bacterial DNA. The efficiency of PCR amplification for each gene was calculated using the standard curve method, E = 10^(− 1/slope)^ − 1.

To each PCR reaction, 5 μl of SYBR Green (Maxima SYBR Green qPCR Master Mix, Thermofisher Scientific TM), 1 μl of each primer (concentration of 5 pmol for Reverse and 10 pmol for Forward), 1 μl of DNA, and 2 μl of DNase/RNase-free water were added to a final volume of 10 μl. Each reaction was performed in duplicate.

The analysis of the qPCR amplification was performed with the Rotor-Gene Q equipment (QIAGEN®, Germany). The samples were processed under the following amplification conditions: an initial thermal denaturation cycle of 5 min at 95 °C, alignment with 30 cycles at 59 °C for 15 s and elongation for 20 s at 72 °C. The conditions were the same for the three pairs of primers used (Universal, *Bacteroidetes* and *Firmicutes*). The expression analysis was carried out by quantifying the relative abundance units (RAU) of *Firmicutes* and *Bacteroidetes* with the formula RAU = 2^-∆Ct^ where: RAU = Relative Abundance Units and ∆Ct = Ct specific primers-Ct universal primers [75].

### Hematic biometry

The quality control was performed through 3 controls: low, normal, and high (KX-21 N SYSMEX LOT: 2R0301) of whole blood to determine haemoglobin, erythrocytes and leukocytes (lymphocytes, MXD and neutrophils) in the automated KX-21 N equipment. Each blood differential of a complete red series and white series grouping (Lymphocytes, neutrophils, and the sum of basophils and eosinophils monocytes (MXD)) were performed.

### Statistical analysis

A descriptive analysis of the variables studied of the 284 participants was performed. For all continuous values, normality hypotheses were evaluated using the Kolmogorov-Smirnov test. The quantitative variables were summarized in terms of means and standard deviation or median and interquartile range [25–75], and the qualitative variables were summarized in frequencies and proportions. Non-parametric U-Mann-Whitney tests were used for comparisons between the medians of the two groups. To group the participants into two equal groups the *Firmicutes/Bacteroidetes* ratio, was operationalized by taking the cut-off point above and below the median of the RAU on each phylum. A chi-square test (×^2^) or Fisher’s exact test was applied for the bivariate analysis of qualitative variables. The odds ratio (OR) and 95% confidence interval (CI) were estimated for measuring the association between *Blastocystis* and the *Firmicutes / Bacteroidetes* ratio. A *p* value < 0.05 was considered significant. The statistical analysis was performed using the Stata® Statistics Package, version 13.0 and Graphpad Prism Software, L.L.C.Version 9.2.0.

## Supplementary Information

**Additional file 1: Figure S1.** A) Relative Abundance Units of *Firmicutes* (F), *Bacteroidetes* (B) and *Firmicutes / Bacteroidetes* ratio (*F / B*) in non-carriers and carriers by *Blastocystis* of the FACSA cohort and UNEME cohort. B) Comparison between FACSA and UNEME subjects non-carriers (NC) and carriers (C) of *Blastocystis* ST-1 using the Mann-Whitney test. Significant difference is shown between ST-1 non-carriers vs carriers FACSA cohort and carriers of both cohorts. C) Comparison between FACSA and UNEME subjects non-carriers (NC) and carriers (C) of *Blastocystis* ST-2 using the Mann-Whitney test. A significant difference was found in the sample of FACSA carriers and non-carriers. D) Comparison between FACSA and UNEME subjects non-carriers (NC) and carriers (C) of *Blastocystis* ST-3 using the Mann-Whitney test. No significant differences were found for this subtype in FACSA or UNEME sample. E) Comparison between FACSA and UNEME subjects non-carriers (NC) and carriers (C) of *Blastocystis* ST-4 using the Mann-Whitney test. Significant differences were found for this subtype in FACSA cohort, and carrier of both cohorts. F) Comparison between FACSA and UNEME subjects non-carriers (NC) and carriers (C) of *Blastocystis* ST-5 using the Mann-Whitney test. No significant differences were found in the cohorts. G) Comparison between FACSA and UNEME non-carriers (NC) and carriers (C) of *Blastocystis* ST-7 using the Mann-Whitney test. A significant difference was found in the FACSA cohort in no carriers’ vs carriers and between the carriers of both cohorts. *p < 0.05.

**Additional file 2: Table S7.** Prevalence of *Blastocystis* subtypes and its association with age, sex, and Hematic Biometry in FACSA cohort.

**Additional file 3: Table S2.** Prevalence of *Blastocystis* and subtypes and their asociation with abdominal pain in FACSA cohort.

**Additional file 4: Table S3.** Prevalence of Blastocystis and subtypes and their asociation with abdominal constipation in FACSA cohort.

**Additional file 5: Table S4.** Prevalence of Blastocystis and subtypes and their association with abdominal pain in a UNEME.

**Additional file 6: Table S5.** Prevalence of Blastocystis and subtypes and their su association with abdominal constipation in UNEME cohort.

**Additional file 7: Table S6.** Characterization of the subjects by geographical area, normal weight and obesity according to *Blastocystis* and subtypes.

**Additional file 8: Table S1.** Primer sequences for genotyping *Blastocystis*.

**Additional file 9: Table S8.** Prevalence of *Blastocystis* subtypes and its association with age, sex, Hematic Biometry and gut microbiota in UNEME cohort.

## Data Availability

The nucleotide sequences generated in the present study have been deposited in GenBank (https://www.ncbi.nlm.nih.gov/) under accession numbers: MZ351752-57.

## Abbreviations

RAU: Relative abundance units
RAUF: Relative abundance units of *Firmicutes*
RAUB: Relative abundance units of *Bacteroidetes*
ratio *F/B*: Ratio *Firmicutes/Bacteroidetes*
IBD: Inflammatory bowel disease
T2D: Type 2 diabetes
CVD: Cardiovascular disease
BMI: Body mass index
FACSA: Facultad de Ciencias de la Salud
UNEME: Unidades en especialidades médicas
UNEME-EC: Unidades en especialidades médicas en enfermedades crónicas
mm^3^: Cubic milimetre
mg/dL: Milligrams per deciliter
mt^2^: Square meter
DNase: Deoxyribonuclease
RNase: Ribonuclease
USA: United States of America
RAPD: Random Amplified Polymorphic acid deoxyribonucleic
PCR: Polymerase Chain Reaction
qPCR: Quantitative polymerase chain reaction
rRNA: Ribosomal ribonucleic acid
ST1: Subtype 1
ST2: Subtype 2
ST3: Subtype 3
ST4: Subtype 4
ST5: Subtype 5
ST7: Subtype 7
IR: Interquartile range
OR: Odds Ratio
CI 95%: Confidence interval to 95%
n: Number
%: Percentage
CHS: Colonic Hypersensitivity
SCFAs: Short-Chain-Fatty-Acids
AOX: Alternativa oxidasa
MXD: Sum of Basophils eosinophils and monocytes
