# Supplementary material for: Prevalence of Blastocystis and its association with Firmicutes/Bacteroidetes ratio in clinically healthy and metabolically ill subjects
Source: BMC Microbiol. 2021 Dec 11;21:339. doi: 10.1186/s12866-021-02402-z (PMC8665487; doi:10.1186/s12866-021-02402-z)
Supplement: Supplementary file 1 — Additional file 1: Figure S1. A) Relative Abundance Units of Firmicutes (F), Bacteroidetes (B) and Firmicutes / Bacteroidetes ratio (F / B) in non-carriers and carriers by Blastocystis of the FACSA cohort and UNEME cohort. B) Comparison between FACSA and UNEME subjects non-carriers (NC) and carriers (C) of Blastocystis ST-1 using the Mann-Whitney test. Significant difference is shown between ST-1 non-carriers vs carriers FACSA cohort and carriers of both cohorts. C) Comparison between FACSA and UNEME subjects non-carriers (NC) and carriers (C) of Blastocystis ST-2 using the Mann-Whitney test. A significant difference was found in the sample of FACSA carriers and non-carriers. D) Comparison between FACSA and UNEME subjects non-carriers (NC) and carriers (C) of Blastocystis ST-3 using the Mann-Whitney test. No significant differences were found for this subtype in FACSA or UNEME sample. E) Comparison between FACSA and UNEME subjects non-carriers (NC) and carriers (C) of Blastocystis ST-4 using the Mann-Whitney test. Significant differences were found for this subtype in FACSA cohort, and carrier of both cohorts. F) Comparison between FACSA and UNEME subjects non-carriers (NC) and carriers (C) of Blastocystis ST-5 using the Mann-Whitney test. No significant differences were found in the cohorts. G) Comparison between FACSA and UNEME non-carriers (NC) and carriers (C) of Blastocystis ST-7 using the Mann-Whitney test. A significant difference was found in the FACSA cohort in no carriers’ vs carriers and between the carriers of both cohorts. *p < 0.05. [file 12866_2021_2402_MOESM1_ESM.docx]

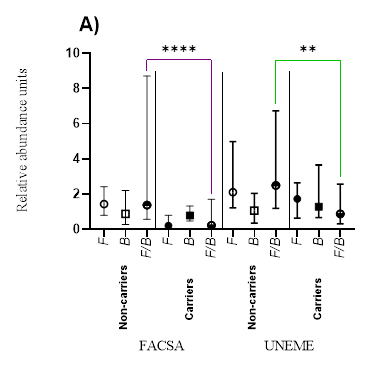

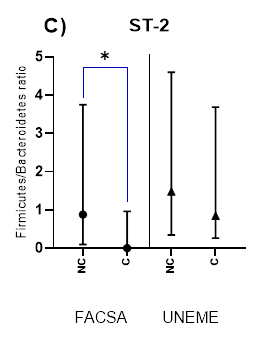

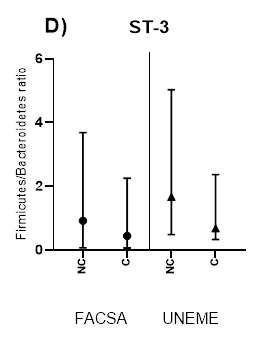

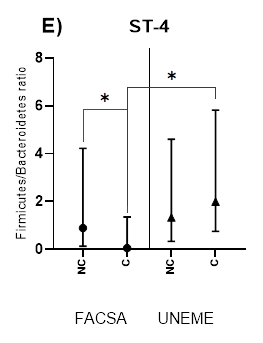

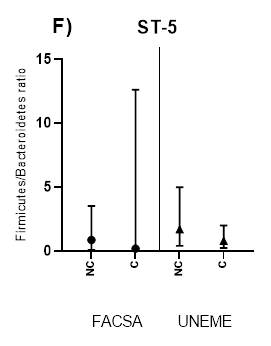

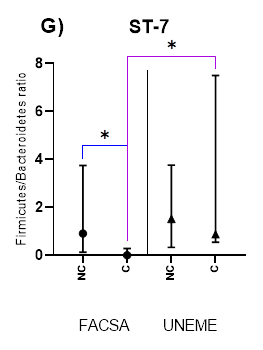

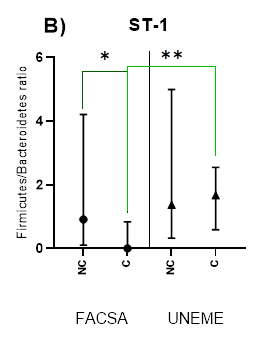


**Figure S1 A)** Relative Abundance Units of *Firmicutes* (F), *Bacteroidetes* (B) and *Firmicutes / Bacteroidetes* ratio (*F / B*) in non-carriers and carriers by *Blastocystis* of the FACSA cohort and UNEME cohort. **B)** Comparison between FACSA and UNEME subjects non-carriers (NC) and carriers (C) of *Blastocystis* ST-1 using the Mann-Whitney test. Significant difference is shown between ST-1 non-carriers vs carriers FACSA cohort and carriers of both cohorts. **C)** Comparison between FACSA and UNEME subjects non-carriers (NC) and carriers (C) of *Blastocystis* ST-2 using the Mann-Whitney test. A significant difference was found in the sample of FACSA carriers and non-carriers. **D)** Comparison between FACSA and UNEME subjects non-carriers (NC) and carriers (C) of *Blastocystis* ST-3 using the Mann-Whitney test. No significant differences were found for this subtype in FACSA or UNEME sample. **E)** Comparison between FACSA and UNEME subjects non-carriers (NC) and carriers (C) of *Blastocystis* ST-4 using the Mann-Whitney test. significant differences were found for this subtype in FACSA cohort, and carrier of both cohorts. **F)** Comparison between FACSA and UNEME subjects non-carriers (NC) and carriers (C) of *Blastocystis* ST-5 using the Mann-Whitney test. No significant differences were found in the cohorts. **G)** Comparison between FACSA and UNEME non-carriers (NC) and carriers (C) of *Blastocystis* ST-7 using the Mann-Whitney test. A significant difference was found in the FACSA cohort in no carriers’ vs carriers and between the carriers of both cohorts. **p*<0.05

Figure S6. 1

Figure S6. 2
