# Supplementary material for: Prevalence of Blastocystis and its association with Firmicutes/Bacteroidetes ratio in clinically healthy and metabolically ill subjects
Source: BMC Microbiol. 2021 Dec 11;21:339. doi: 10.1186/s12866-021-02402-z (PMC8665487; doi:10.1186/s12866-021-02402-z)
Supplement: Supplementary file 2 — Additional file 2: Table S7. Prevalence of Blastocystis subtypes and its association with age, sex, and Hematic Biometry in FACSA cohort. [file 12866_2021_2402_MOESM2_ESM.docx]

Table S7. Prevalence of *Blastocystis* subtypes and its association with age, sex, and Hematic Biometry in FACSA cohort.

|  | **ST1** | | **ST2** | | **ST3** | | **ST4** | | **ST5** | | **ST7** | |
| --- | --- | --- | --- | --- | --- | --- | --- | --- | --- | --- | --- | --- |
|  | no carriers | carriers | no carriers | carriers | no carriers | carriers | no carriers | carriers | no carriers | carriers | no carriers | carriers |
| Age, median IR | 20  (19-21) | 20.5  (20-21) | 20  (19-21) | 19.5  (17.5-21) | 20  (19-21) | 20  (19-21.5) | 20  (19-21) | 20  (19-21) | 20  (19-21) | 20  (20-21) | 20  (19-21) | 20  (19.5-21) |
| Sex, n % |  |  |  |  |  |  |  |  |  |  |  |  |
| Male | 64  (94.12) | 4  (5.88) | 65  (95.59) | 3  (4.41) | 58  (85.29) | 10  (14.71) | 61  (89.71) | 7  (10.29) | 68  (100) | 0  (0) | 62 (91.18) | 6  (8.82) |
| Female | 122  (92.42) | 10  (7.58) | 127  (96.21) | 5  (3.79) | 114 (86.36) | 28  (14) | 123 (93.18) | 9  (6.82) | 127  (96.21) | 5  (3.79) | 126 (95.45) | 6 (4.55) |
| BMI, n % |  |  |  |  |  |  |  |  |  |  |  |  |
| Normal | 105  (94.59) | 6  (5.41) | 107  (96.40) | 4  (3.60) | 100 (90.09) | 11  (9.91) | 99  (89.19) | 12  (10.81) | 108  (97.30) | 3  (2.70) | 105 (94.59) | 6  (5.41) |
| Overw/obesity | 81  (91.01) | 8  (8.99) | 85  (95.51) | 4  (4.49) | 72  (80.90) | 17  (19.10) | 85  (95.51) | 4  (4.49) | 87  (97.75) | 2  (2.25) | 83 (93.26) | 6  (6.74) |
| Place of residence, n % |  |  |  |  |  |  |  |  |  |  |  |  |
| Urban | 156  (91.76) | 14  (8.24) | 162  (95.29) | 8  (4.71) | 145 (85.29) | 25  (14.71) | 158  (92.94) | 12  (7.06) | 165  (97.06) | 5  (2.94) | 162 (95.29) | 8  (4.71) |
| Rural | 30  (100) | 0  (0) | 30  (100) | 0  (0) | 27  (90) | 3  (10) | 26  (86.67) | 4  (13.33) | 30  (100) | 0  (0) | 26 (86.67) | 4  (13.33) |
| Hematic Biometry, median IR |  |  |  |  |  |  |  |  |  |  |  |  |
| Leukocytes/mm3 | 6.5  (5.4-7.8) | 6.55  (5.8-8.4) | 6.5  (5.45-7.75) | 7.1  (4.45-7.95) | 6.5  (5.4-7.75) | 6.65  (5.45-8.05) | **6.5**  **(5.4-7.8)** | **6**  **(5.5.7.9)*** | 6.5  (5.4-7.8) | 6.2  (5.8-6.3) | 6.5  (5.4-7.7) | 7.05  (5.5-8.3) |
| Total Lymphocytes/  mm3 | 2.1  (1.7-2.5) | 2.2  (1.7-2.5) | 2.1  (1.7-2.5) | 2.2  (1.7-2.5) | 2.1  (1.7-2.5) | 2.2  (1.8-2.6) | 2.1  (1.7-2.5) | 2.2  (1.8-2.9) | **2.1**  **(1.7-2.5)** | **2.7**  **(2.5-2.75)*** | 2.2  (1.7-2.5) | 1.9  (1.8-2.4) |
| TotalMXD/mm3 | 0.5  (0.4-0.6) | 0.5  (0.4-0.6) | 0.5  (0.4-0.6) | 0.4  (0.4-0.6) | 0.5  (0.4-0.6) | 0.5  (0.4-0.7) | 0.5  (0.4-0.6) | 0.5  (0.3-0.6) | 0.5  (0.4-0.6) | 0.55  (0.5-0.8) | 0.5  (0.4-0.6) | 0.6  (0.4-0.7) |
| Total Neutrophils/mm3 | 3.6  (3-4.7) | 3.95  (2.9-5) | 3.7  (2.9-4.7) | 4.2  (5 3.5-5) | 3.6  (2.95-4.75) | 3.7  (3-4.7) | 3.7  (2.9-4.8) | 3.2  (3-4.2) | 3.7  (3-4.8) | 2.95 (2.75-3.15) | 3.7  (3-4.7) | 3.6  (2.7-5.5) |
| Platelets | 246.5  (220-282) | 250  (227-285) | 246  (218.5-281.5) | 258  (244-293) | 248 (221.5-284.5) | 236.5 (207.5-270) | 247  (221-282) | 229  (185-287) | 247  (220-285) | 243  (226-277) | 247 (218.5-283.5) | 246.5  (223.5-276) |

IR: interquartil Rank, n: number; %; percentage; mm3: cubic millimeter. Overw/obesity; overweight and obesity; MXD: monocyte, eosinophil, and basophil count; mm3: cubic millimeter Mann-Whitney-Wilcoxon; x^2^: chi-square; **p*<0.05.
