# Supplementary material for: Prevalence of Blastocystis and its association with Firmicutes/Bacteroidetes ratio in clinically healthy and metabolically ill subjects
Source: BMC Microbiol. 2021 Dec 11;21:339. doi: 10.1186/s12866-021-02402-z (PMC8665487; doi:10.1186/s12866-021-02402-z)
Supplement: Supplementary file 3 — Additional file 3: Table S2. Prevalence of Blastocystis and subtypes and their asociation with abdominal pain in FACSA cohort. [file 12866_2021_2402_MOESM3_ESM.docx]

Table S2 Prevalence of *Blastocystis* and subtypes and their asociation with abdominal dolor in FACSA cohort.

|  | n (%) | OR | CI 95 % | *P value* |
| --- | --- | --- | --- | --- |
| *Blastocystis* | 40 (42.5) | 0.63 | 0.35-1.13 | 0.12 |
| ST1 | 2 (5.0) | **0.15** | **0.03-0.71** | **0.01** |
| ST2 | 5 (12.5) | 1.61 | 0.37-6.94 | NS |
| ST3 | 14 (35.0) | 1.22 | 0.48-2.58 | NS |
| ST4 | 6 (15.0) | 0.53 | 0.18-1.54 | NS |
| ST5 | 4(10.0) | 3.91 | 0.42-35.68 | NS |
| ST7 | 5(12.5) | 0.65 | 0.20-2.15 | NS |

n: number; OR: Odds ratio; CI: confidence interval.
