# Supplementary material for: Prevalence of Blastocystis and its association with Firmicutes/Bacteroidetes ratio in clinically healthy and metabolically ill subjects
Source: BMC Microbiol. 2021 Dec 11;21:339. doi: 10.1186/s12866-021-02402-z (PMC8665487; doi:10.1186/s12866-021-02402-z)
Supplement: Supplementary file 4 — Additional file 4: Table S3. Prevalence of Blastocystis and subtypes and their asociation with abdominal constipation in FACSA cohort. [file 12866_2021_2402_MOESM4_ESM.docx]

Table S3. Prevalence of Blastocystis and subtypes and their asociation with abdominal constipation in FACSA cohort.

|  | n (%) | OR | CI 95 % | *P value* |
| --- | --- | --- | --- | --- |
| *Blastocystis* | 49 (43.7) | 0.63 | 0.35-1.16 | 0.14 |
| ST1 | 7 (14.2) | 0.72 | 0.23-2.24 | NS |
| ST2 | 6 (12.2) | 1.92 | 0.38-9.95 | NS |
| ST3 | 17 (34.6) | 1.32 | 0.51-2.93 | NS |
| ST4 | 6 (12.2) | **0.34** | **0.11-0.99** | **0.04** |
| ST5 | 5(10.20) | / | / | NS |
| ST7 | 8(13.6) | 1.28 | 0.37-4.44 | NS |

n: numer; OR: Odds ratio; CI: confidence interval.
