# Supplementary material for: Prevalence of Blastocystis and its association with Firmicutes/Bacteroidetes ratio in clinically healthy and metabolically ill subjects
Source: BMC Microbiol. 2021 Dec 11;21:339. doi: 10.1186/s12866-021-02402-z (PMC8665487; doi:10.1186/s12866-021-02402-z)
Supplement: Supplementary file 5 — Additional file 5: Table S4. Prevalence of Blastocystis and subtypes and their association with abdominal pain in a UNEME. [file 12866_2021_2402_MOESM5_ESM.docx]

Table S4. Prevalence of Blastocystis and subtypes and their association with abdominal pain in a UNEME.

|  | n (%) | OR | CI 95 % | *P value* |
| --- | --- | --- | --- | --- |
| *Blastocystis* | 18 (64.2) | 1 | 0.36-2.71 | NS |
| ST1 | 3 (16.6) | 0.86 | 0.18-3.94 | NS |
| ST2 | 2 (11.1) | 0.97 | 0.15-6.24 | NS |
| ST3 | 6 (33.3) | 1.32 | 0.39-4.46 | NS |
| ST4 | 1 (5.5) | 0.46 | 0.04-4.75 | NS |
| ST5 | 5(27.7) | 0.77 | 0.22-2.61 | NS |
| ST7 | 1(5.5) | 0.26 | 0.02-2.41 | NS |

N: number; OR: Odds ratio; CI: confidence interval.
