# Supplementary material for: Prevalence of Blastocystis and its association with Firmicutes/Bacteroidetes ratio in clinically healthy and metabolically ill subjects
Source: BMC Microbiol. 2021 Dec 11;21:339. doi: 10.1186/s12866-021-02402-z (PMC8665487; doi:10.1186/s12866-021-02402-z)
Supplement: Supplementary file 6 — Additional file 6: Table S5. Prevalence of Blastocystis and subtypes and their su association with abdominal constipation in UNEME cohort. [file 12866_2021_2402_MOESM6_ESM.docx]

Table S5. Prevalence of Blastocystis and subtypes and their su association with abdominal constipation in UNEME cohort

|  | n (%) | OR | CI 95 % | *P value* |
| --- | --- | --- | --- | --- |
| *Blastocystis* | 32 (69.5) | 1.52 | 0.60-3.83 | NS |
| ST1 | 5 (16.13) | 1.33 | 0.29-6.00 | NS |
| ST2 | 3 (9.6) | 1.17 | 0.18-7.46 | NS |
| ST3 | 9 (29.0) | 1.50 | 0.45-4.95 | NS |
| ST4 | 4 (12.9) | 3.31 | 0.35-31.09 | NS |
| ST5 | 7 (22.5) | 0.62 | 0.20-1.92 | NS |
| ST7 | 3 (9.6) | 0.34 | 0.04-1.49 | 0.15 |

n: number; OR: Odds ratio; CI: confidence interval.
