# Supplementary material for: Prevalence of Blastocystis and its association with Firmicutes/Bacteroidetes ratio in clinically healthy and metabolically ill subjects
Source: BMC Microbiol. 2021 Dec 11;21:339. doi: 10.1186/s12866-021-02402-z (PMC8665487; doi:10.1186/s12866-021-02402-z)
Supplement: Supplementary file 7 — Additional file 7: Table S6. Characterization of the subjects by geographical area, normal weight and obesity according to Blastocystis and subtypes. [file 12866_2021_2402_MOESM7_ESM.docx]

| **VARIABLE** | ***Blastocystis*** | **ST1** | **ST2** | **ST3** | **ST4** | **ST5** | **ST7** |
| --- | --- | --- | --- | --- | --- | --- | --- |
| n (%) | 149 (52.46 %) | 22 (14.86 %) | 13 (8.78 %) | 42 (28.28 %) | 21 (14.19 %) | 21 (14.19 %) | 21 (14.19 %) |
| **sex, n(%)** |  |  |  |  |  |  |  |
| Male n(%) | 42 (50.60 %) | 5 (11.90%) | 3 (7.14 %) | 13 (30.95 %) | 7 (16.67 %) | 4 (9.52 %) | 8 (19.05 %) |
| Female (%) | 107 (53.23 %) | 17 (16.04%) | 10 (9.43 %) | 29 (27.36 %) | 14(13.21 %) | 17 (16.04 %) | 13 (12.26 %) |
|  |  |  |  |  |  |  |  |
| Age Median (IR) | 21 (19-45 ) | 21.5 (20-53) | 21 (19-62) | 21.5 (20-56) | 21 (19-22) | **49 (23-64)** | 21 (20-55) |
|  |  |  |  |  |  |  |  |
| BMI Median (IR) | 26.3 (22.2-31.4) | 26(22.8-30.0) | 25.6 (23.8-30.5) | 27.2(23.6-30.6) | **22.1(19.9-39.7)** | 28.6(25.3-34.2) | 28.85(24-33.8) |
|  |  |  |  |  |  |  |  |
|  |  |  |  |  |  |  |  |
| **Place of reside** |  |  |  |  |  |  |  |
| rural n (%) | 52 (34.90 %) | 5 (9.80 %) | 5 (9.80 %) | **14 (27.45 %)** | 7 (13.73 %) | **12 (23.53 %)** | 9 (17.65 %) |
| urban n (%) | 97 (65.10 %) | 17(17.53 %) | 8 (8.25 %) | 28 (28.87 %) | 14(14.43 %) | 9 (9.28 %) | 12 (12.37 %) |
|  |  |  |  |  |  |  |  |
|  |  |  |  |  |  |  |  |
| **Obesity n(%)** |  |  |  |  |  |  |  |
| Obeso n (%) | 92 (61.74 %) | 14 (15.38 %) | 8 (8.79%) | **29 (31.87 %)** | 9 (9.89 %) | 16 (17.58%) | 12 (13.19 %) |
| Not obeso n (%) | 57 (38.26 %) | 8 (14.04 %) | 5 (8.77 %) | **13 (22.81 %)** | **12 (21.05 %)** | 5 (8.77 %) | 9 (15.79 %) |

Table S6: Characterization of the subjects by geographical area, normal weight and obesity according to *Blastocystis* and subtypes

n: number; IR: interquartil range; BMI: Body mass index.
