# Supplementary material for: Prevalence of Blastocystis and its association with Firmicutes/Bacteroidetes ratio in clinically healthy and metabolically ill subjects
Source: BMC Microbiol. 2021 Dec 11;21:339. doi: 10.1186/s12866-021-02402-z (PMC8665487; doi:10.1186/s12866-021-02402-z)
Supplement: Supplementary file 8 — Additional file 8: Table S1. Primer sequences for genotyping Blastocystis. [file 12866_2021_2402_MOESM8_ESM.docx]

Table S1. Primer sequences for genotyping *Blastocystis*.

| Subtype | STS | Product  (pb) | Primer sequence | Gen Bank accession | Clade in the SSU rRNA Phylogeny |
| --- | --- | --- | --- | --- | --- |
| 1 | SB82 | 462 | F GAAGGACTCTCTGACGATGA  R GTCCAAATGAAAGGCAGC | AF166086 | I |
| 2 | SB155 | 650 | F ATCAGCCTACAATCTCCTC  R ATCGCCACTTCTCCAAT | AF166087 | VII |
| 3 | SB227 | 526 | F AGGATTTGGTGTTTGGAGA  R TTAGAAGTGAAGGAGATGGAAG | AF166088 | III |
| 3 | SB228 | 473 | F GAC TCC AGA AAC TCG CA  R TCT TGT TTC CCC AGT TAT CC | AF166089 | III |
| 3 | SB229 | 631 | F-CACTGTGTCGTCATTGTTTTG  R-AGGGCTGCATAATAGAGTGG | AF166090 | III |
| 4 | SB332 | 338 | F GCATCCAGACTACTATCAACATT  R CCATTTTCAGACAACCACTTA | AF166091 | VI |
| 5 | SB340 | 704 | F TGTTCTTGTGTCTTCTCAGCTC  R TTCTTTCACACTCCCGTCAT | AY048752 | II |
| 7 | SB337 | 487 | F GTCTTTCCCTGTCTATTCTGCA  R AATTCGGTCTGCTTCTTCTG | AY048750 | IV |

Mohamed, R. T., El-bali, M. A., Mohamed, A. A., Abdel-fatah, M. A., El-malky, M. A., Mowafy, N. M., … Al-harthi, S. A. (2017). Subtyping of Blastocystis sp . isolated from symptomatic and asymptomatic individuals in Makkah , Saudi Arabia, 1–7. https://doi.org/10.1186/s13071-017-2114-8
