# Supplementary material for: Prevalence of Blastocystis and its association with Firmicutes/Bacteroidetes ratio in clinically healthy and metabolically ill subjects
Source: BMC Microbiol. 2021 Dec 11;21:339. doi: 10.1186/s12866-021-02402-z (PMC8665487; doi:10.1186/s12866-021-02402-z)
Supplement: Supplementary file 9 — Additional file 9: Table S8. Prevalence of Blastocystis subtypes and its association with age, sex, Hematic Biometry and gut microbiota in UNEME cohort. [file 12866_2021_2402_MOESM9_ESM.docx]

Table S8. Prevalence of *Blastocystis* subtypes and its association with age, sex, Hematic Biometry and gut microbiota in UNEME cohort.

|  | **ST1** | | **ST2** | | **ST3** | | **ST4** | | **ST5** | | **ST7** | |
| --- | --- | --- | --- | --- | --- | --- | --- | --- | --- | --- | --- | --- |
|  | no carriers | carriers | no carriers | carriers | no carriers | carriers | no carriers | carriers | no carriers | carriers | no carriers | carriers |
| Age, median IR | 55  (47-63) | 60  (49-68) | **53**  **(46-63)** | **63**  **(62-64)*** | **53**  **(46-63)** | **60**  **(56-63)*** | 54  (47-63) | 57  (57-62) | 55  (47-61) | 62  (45.5-70) | 54  (46-63) | 57  (53-58) |
| Sex, n % |  |  |  |  |  |  |  |  |  |  |  |  |
| Male | 14  (93.33) | 1  (6.67) | 15  (100) | 0  (0) | 12  (80) | 3  (20) | 15  (100) | 0  (0) | 11  (73.33) | 4  (26.67) | 13  (86.67) | 2  (13.33) |
| Female | 61  (89.71) | 7  (10.29) | 63 (92.65) | 5  (7.35) | 57  (83.82) | 11  16.18) | 63 (92.65) | 5  (7.35) | 56  (82.35) | 12 (17.65) | 61  (89.71) | 7  (10.29) |
| BMI, n % |  |  |  |  |  |  |  |  |  |  |  |  |
| Normal | 5  (71.43) | 2  (28.57) | 6  (85.71) | 1  (14.29) | 5  (71.43) | 2  (28.57) | 7  (100) | 0  (0) | 5  (71.43) | 2  (28.57) | **4**  **(57.14)** | **3**  **(42.86)*** |
| Overw/obesity | 70  (92.11) | 6  (7.89) | 72  (94.74) | 4  (5.26) | 64  (84.21) | 12 (15.79) | 71  (93.42) | 5  (6.58) | 62  (81.58) | 14 (18.42) | **70**  **(92.11)** | **6**  **(7.89)*** |
| Place of residence, n % |  |  |  |  |  |  |  |  |  |  |  |  |
| Urban | 24  (88.89) | 3  (11.11) | 27  (100) | 0  (0) | 24  (88.89) | 3  (11.11) | 25  (92.59) | 2  (7.41) | 23  (85.19) | 4  (14.81) | 23  (85.19) | 4  (14.81) |
| Rural | 51  (91.07) | 5  (8.93) | 51  (91.07) | 5  (8.93) | 45  (80.36) | 11  (14.69) | 53  (94.64) | 3  (5.36) | 44  (78.57) | 12 (21.43) | 51  (91.07) | 5  (8.93) |
| Hematic Biometry, median IR |  |  |  |  |  |  |  |  |  |  |  |  |
| Leukocytes/mm3 | 7.4  (6.3-8.4) | 7.95  (5.4-9.55) | 7.65  (6.3-8.7) | 6.8  (6-7.3) | 7.3  (6.3-8.7) | 7.75  (7.1-8.2) | 7.75  (6.7-8.7) | 6.3  (5.3-7) | 7.3  (6.3-8.7) | 7.9  (6.95-8.55) | 7.35  (6.3-8.4) | 8.2  (7.3-9.4) |
| Total Lymphocytes/mm3 | 2.4  (2-2.7) | 2.15  (1.85-2.45) | 2.4  (2-2.7) | 2.2  (2.2-2.4) | 2.3  (2-2.7) | 2.6  (2.1-2.9) | 2.4  (2-2.7) | 2.1  (1.45-2.55) | 2.4  (2-2.7) | 2.2  (2.1-2.9) | 2.35  (2-2.7) | 2.4  (2.1-2.5) |
| TotalMXD/mm3 | 0.4  (0.2-0.7) | 0.6  (0.5-0.7) | 0.5  (0.3-0.7) | 0.2  (0.15-0.45) | 0.5  (0.2-0.6) | 0.65  (0.3-0.8) | 0.5  (0.3-0.7) | 0.5  (0.2-0.7) | 0.5  (0.25-0.65) | 0.5  (0.3-0.8) | **0.4**  **(0.2-0.6)** | **0.95**  **(0.65-1.25)*** |
| Total Neutrophils/mm3 | 4.6 (3.8-5.4) | 3.95  (2.75-5.9) | 4.6  (3.8-5.4) | 4.7  (3.75-5.4) | 4.5  (3.5-5.2) | 4.9  (4.3-5.9) | 4.7  (3.8-5.4) | 3.9  (3.1-5.9) | 4.5  (3.65-5.75) | 4.9  (4.5-5.1) | 4.5  (3.8-5.4) | 4.95  (4.35-6.6) |
| Platelets | 262  (234-316) | 219  (179-295) | 261  (222-316) | 259  (249-284) | 257 (222-316) | 271  (246-312) | 261  (227-312) | 257  (204-340) | 261  (234-312) | 255  (216.5-328.5) | **255.5 (220-299)** | **329**  **(299-349)*** |

IR: interquartil Rank, n: number; %; percentage; mm3: cubic millimeter. Overw/obesity; overweight and obesity; MXD: monocyte, eosinophil, and basophil count; mm3: cubic millimeter Mann-Whitney-Wilcoxon; x^2^: chi-square; **p*<0.05.
